# An Event-Specific Real-Time PCR Method for Measuring Transgenic Lysozyme Goat Content in Trace Samples

**DOI:** 10.3390/foods10050925

**Published:** 2021-04-23

**Authors:** Wenting Xu, Jinjie Cui, Biao Liu, Litao Yang

**Affiliations:** 1 Joint International Research Laboratory, Metabolic and Developmental Sciences, School of Life Sciences and Biotechnology, Shanghai Jiao Tong University, Shanghai 200240, China; hope1xu@163.com; 2 State Key Laboratory, Cotton Biology, Institute of Cotton Research, Chinese Academy of Agricultural Sciences, Anyang 455000, China; cuijinjie@126.com; 3 Key Laboratory on Biosafety, Nanjing Institute of Environmental Sciences, Ministry of Ecology and Environment, Nanjing 210042, China; liubiao@nies.org

**Keywords:** event-specific real-time quantitative polymerase chain reaction, transgenic human lysozyme, transgenic goat, goat milk, trace samples

## Abstract

Lysozymes are used in sterilisation, antisepsis, dairy additives, inflammation, and cancer. One transgenic goat line expressing high levels of human lysozyme (hLZ) in goat milk has been developed in China. Herein, we established an event-specific real-time polymerase chain reaction (real-time PCR) method to detect the transgenic hLZ goat line. The developed method has high specificity, sensitivity and accuracy, and a wide quantitative dynamic range. The limit of detection and limit of quantification was 5 and 10 copies per reaction, respectively. The practical sample analysis results showed that the method could identify and quantify transgenic lysozyme content in trace samples in routine lab analyses. Furthermore, the potential applicability in risk assessment, such as molecular characterisation and gene horizontal transfer, was confirmed. We believe that this method is suitable for the detection of transgenic hLZ goat line and its derivate.

## 1. Introduction

Transgenic animals are generated by introducing foreign genes into fertilised eggs or embryos of recipient animals through genetic engineering methodologies, in which foreign genes can be stably integrated into the animal genome and be passed on to their offspring [1]. With continual improvement in recombinant DNA techniques, more and more transgenic animals are being approved for commercialisation. In 2010, a human recombinant C1 esterase inhibitor (Ruconest) purified from transgenic rabbit milk produced by the Dutch Pharming Company was approved by the European Medicines Agency (EMA) [2]. In 2015, transgenic salmon harbouring a growth hormone gene were approved for use in food materials [3]. However, the public still have concerns about the safety of transgenic animals and their products, with particular emphasis on the escape (release) of transgenic animals and their impact on the environment, horizontal gene transfer, and food safety [4]. Regarding the above safety aspects, the development of sensitive detection methods for transgenes is essential. In addition, most countries around the world have established genetically modified organism (GMO) labelling systems in which GM food/feeds containing GM components above a specific threshold must be labelled [5]. Therefore, reliable and sensitive GM detection technologies are needed for safety assessment and GM labelling regulation implementation.

In order to establish effective detection methods for transgenic animals and their products, methodologies targeting nucleic acids and proteins are typically used, such as conventional polymerase chain reaction (PCR), multiplex PCR, real-time PCR, digital PCR, enzyme-linked immunosorbent assay (ELISA), and immunochromatographic strips [6,7]. Methods targeting nucleic acids are primarily used for genetically modified organisms (GMOs). According to the specificity of target sequences, nucleic acid detection methods can be divided into four categories: screening-, gene-, construct- and event-specific [8]. In general, screening- and event-specific methods are mainly used in routine lab analysis. Firstly, samples are tested using screening methods targeting against the promoters, terminators, marker genes and reporter genes. Additionally, an event-specific detection method based on flanking sequences is then used for the identification and quantification of transgenic lines when the screening test results are positive [9,10,11]. Currently, real-time PCR is mainly used for transgene detection, such as GM content quantification, genetic stability, copy number determination, and horizontal gene transfer evaluation. For example, event-specific methods for myostatin (MSTN) gene knockout pigs and transgenic overexpressed PBD-2 gene pigs were established based on the determined insert site junction sequences [12,13]. An event-specific multiplex PCR assay was developed to assess six transgenic animal lines (GM human lactoferrin cattle, GM human lysozyme cattle, GM human α-lactalbumin cattle, myostatin knockout pigs, GM phytase pigs, and GM v-3 fatty acid desaturase pigs) [14]. A real-time PCR method specific for the Atlantic salmon (Salmo salar) growth hormone gene (GH1) was developed for the detection and quantification of S. salar L. DNA and Salmonidae ingredients in commercial foods [15]. Osamu et al. (2013) developed a real-time PCR method targeting the *hEpo* gene to test the GM content originating from GM chickens carrying the *hEpo* gene in raw chicken meat and processed foods [16].

Human lysozyme is a natural antibacterial protein in milk, which is commonly used in sterilisation, antisepsis, dairy additives, inflammation/cancer treatment, and other fields [17]. However, natural human lysozyme is difficult to obtain in large quantities, and lysozyme from other species tends to suffer from low activity and side effects, and hence cannot be industrialised [18]. The human *lysozyme* (hLZ) gene was transferred into goats and expressed specifically in the mammary gland, yielding a final protein concentration up to 270 mg/L, 68% of the content in human milk [19]. The transgenic goats harbouring an expression cassette with the sheep β-casein promoter and hLZ gDNA using somatic cell-mediated transgenic cloning were produced, which expressed the human *lysozyme* specifically in the mammary gland in China [20]. The transgenic hLZ goat is in the process of commercialisation. However, there are no suitable methods for detection of the transgenic hLZ goat line.

In this study, we aimed to develop an event-specific real-time PCR method for transgenic hLZ goats, and to detect the GM hLZ goat content in trace samples (goat milk and faeces) and evaluate the exogenous gene inheritance stability among transgenic goat offspring lines.

## 2. Materials and Methods

### 2.1. Materials

The G0 generation female transgenic hLZ gene goat was developed by Shanghai TRANSGENIC Research Center, China, through somatic cell cloning, and F1–F8 generations were produced from the G0 generation [20]. Five goat milk samples were collected from four transgenic hLZ female goats and one non-transgenic female goat with the same breed, parity, and stage of lactation. Goat milk was collected from each half of the udder into sterile tubes during the morning milking by first cleaning the teats with 96% ethanol and discarding the first three streams of milk. Blood samples (10 mL) from G0 transgenic hLZ gene goat lines and non-transgenic goats were collected from the carotid artery to establish the event-specific real-time PCR method. Four standard goat blood samples with different GM contents (S1 = 5.0%, S2 = 2.0%, S3 = 0.9%, S4 = 0.5%) were prepared by mixing transgenic and non-transgenic goat blood samples to evaluate the accuracy and precision of the developed real-time PCR assay. A total of 16 blood samples were collected from offspring (F1–F8) generations of G0 transgenic hLZ goat lines. A total of 18 fresh faecal samples from 16 transgenic and 2 non-transgenic goats were collected shortly after goats were discharged. Five soil samples were collected from the living environment where transgenic goat faeces were deposited. All detailed information for blood, milk and faecal samples is included in Supplementary Table S1. Blood samples from transgenic human *lactoferrin (hLF)* and transgenic human *serum albumin (hSA)* goats were kindly supplied by Shanghai JieLong Biotech Company, China. All animals were housed and cared for under Association for Assessment and Accreditation of Laboratory Animal Care (AAALAC)-approved conditions.

### 2.2. DNA Extraction

DNA from blood, faecal, and soil samples was extracted using commercial DNA extraction kits according to their manuals. DNA was extracted from 200 µL blood or 2 mL milk samples using a TIANamp Genomic DNA Kit (DP304, TIANGEN Biotech, Shanghai, China). DNA was extracted from 500 mg faecal samples using a QIAamp Fast DNA Stool Mini Kit (51604, QIAGEN, Hilden, Germany). DNA was extracted from 500 mg soil samples using a DNeasy PowerSoil Kit (142579, QIAGEN, Hilden, Germany). The quality and quantity of extracted DNA were evaluated using a NanoDrop 2000 (Thermo Fisher Scientific, Waltham Mass, MA, USA) and 1% agarose gel electrophoresis in 0.5 × TBE with ethidium bromide staining. All extracted DNA was stored at 4 °C for further experiments.

### 2.3. Primers and Probes

**Figure 1:**
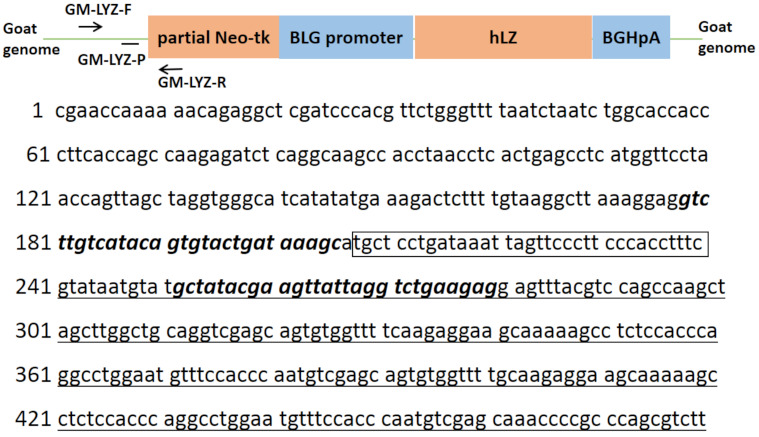
Sequence information and the location of the event-specific primer/probe set for transgenic human lysozyme (hLZ) goat lines. Partial Neo-tk, partial sequence of the neomycin resistance cassette; BLG promoter, bovine β-lactoglobulin promoter; hLZ, human lysozyme gene; BGHpA, bovine growth factor polyadenylation fragment. Lowercase letters not underlined indicate the goat genomic DNA sequence. Lowercase letters underlined indicate exogenous DNA from the partial Neo-tk cassette. Letters in bold italics indicate the designed forward and reverse primers. Letters in the frame indicate the designed TaqMan probe.

TaqMan fluorescent real-time PCR primers and probe for transgenic goat event analysis were designed with Beacon designer software version 8.0 (PREMIER Biosoft, San Francisco, CA, USA) according to the junction sequence surrounding the exogenous DNA integrated site. The location of the event-specific primers/probe set, and the event-specific sequences, are shown in Figure 1. The primers and probe for the goat endogenous prolactin receptor reference gene (GenBank Accession Number AF041979.1) were developed in our previous work [21]. All primers and probes are listed in Table 1 and were purchased from Invitrogen Ltd. (Shanghai, China).

### 2.4. Conventional PCR and Quantitative Real-Time PCR

Conventional PCR was carried out in a final volume of 25 µL, including 12.5 µL Taq PCR Master Mix (Huirui, Shanghai, China), 1 µL of forward primer GM-LYZ-F (10 µM), 1 µL of reverse primer GM-LYZ-R (10 nM), 2 µL of genomic DNA as template, and 8.5 µL of ddH_2_O. PCR amplification was performed using a thermal cycling profile for 5 min at 95 °C, followed by 35 cycles of denaturation at 95 °C for 10 s, annealing at 60 °C for 30 s, and extension at 72 °C for 1 min, followed by a 7 min additional extension step at 72 °C. PCR products were analysed by electrophoresis on 2.0% (*w/v*) agarose gels stained with ethidium bromide for ~30 min at 120 V.

Quantitative real-time PCR was carried out in a final volume of 25 µL, including 12.5 µL HR qPCR Master Mix (Huirui), 1 µL of forward primer (10 µM), 1 µL of reverse primer (10 µM), 0.5 µL of TaqMan probe (10 µM), 5 μL of genomic DNA as template, and 5 µL ddH_2_O. Reactions were performed on an ABI7900 instrument (Applied Biosystems, Foster City, CA, USA) with thermal cycling involving 10 min at 95 °C, followed by 45 cycles at 95 °C for 15 s and 60 °C for 1 min. The fluorescent signal was measured during the extension step of each cycle. Reactions were performed three times in triplicate.

### 2.5. Evaluation of the Performance of the Event-Specific Real-Time PCR Assay

To evaluate the performance of the developed event-specific real-time PCR assay for transgenic hLZ goat analysis, the standard curve, specificity, sensitivity, repeatability, accuracy, and precision were separately assessed. To evaluate the specificity, three other transgenic goat lines (transgenic *hLA*, transgenic *hSA*, and transgenic *Prnp* deletion) were tested. Genomic DNA from transgenic hLZ goats was serially diluted with 0.1 × TE buffer to final concentrations of 160,000, 20,000, 2500, 300, 40 and 5 haploid genomic copies per 5 μL, and these were used as calibrators for standard curve construction. Non-GM goat line DNA and ddH_2_O served as negative and blank controls, respectively. To determine the limit of detection (LOD) and limit of quantification (LOQ), six transgenic hLZ goat DNA gradient dilutions with concentrations corresponding to 2000, 200, 100, 50, 10 and 5 haploid genomic copies per 5 μL were prepared and tested. The repeatability of the established real-time PCR assay was also estimated using the above six dilutions. Reactions at each DNA concentration were repeated three times, in triplicate. Four standard goat blood samples with known GM content (S1–S4) were quantified and used to evaluate the accuracy and precision of the developed real-time PCR assay. The standard curve was constructed by plotting the Ct values measured against the logarithm of the DNA copy number for the calibration points with Microsoft Excel 2016, and these data were composed of a linear regression line. The amount copy numbers of tested samples were calculated according to the Ct values and constructed standard curves. The mean Ct values, standard deviation (SD), relative standard deviation (RSD), and quantified bias were statistically analysed with Microsoft Excel 2016.

### 2.6. Application of the Developed Real-Time PCR Assay in Practical Sample Analysis

After evaluating the performance of the established real-time PCR assay, it was used to measure the GM content in several samples for the risk assessment of transgenic hLZ goat lines. Specifically, 16 blood samples, 5 milk samples, 18 faecal samples, and 5 soil samples from the living environment were tested, all in triplicate. The values of mean Ct, SD, and RSD were statistically analysed with Microsoft Excel 2016.

## 3. Results and Discussion

### 3.1. Specificity of the Event-Specific Real-Time PCR Assay for Transgenic hLZ Goats

**Figure 2:**
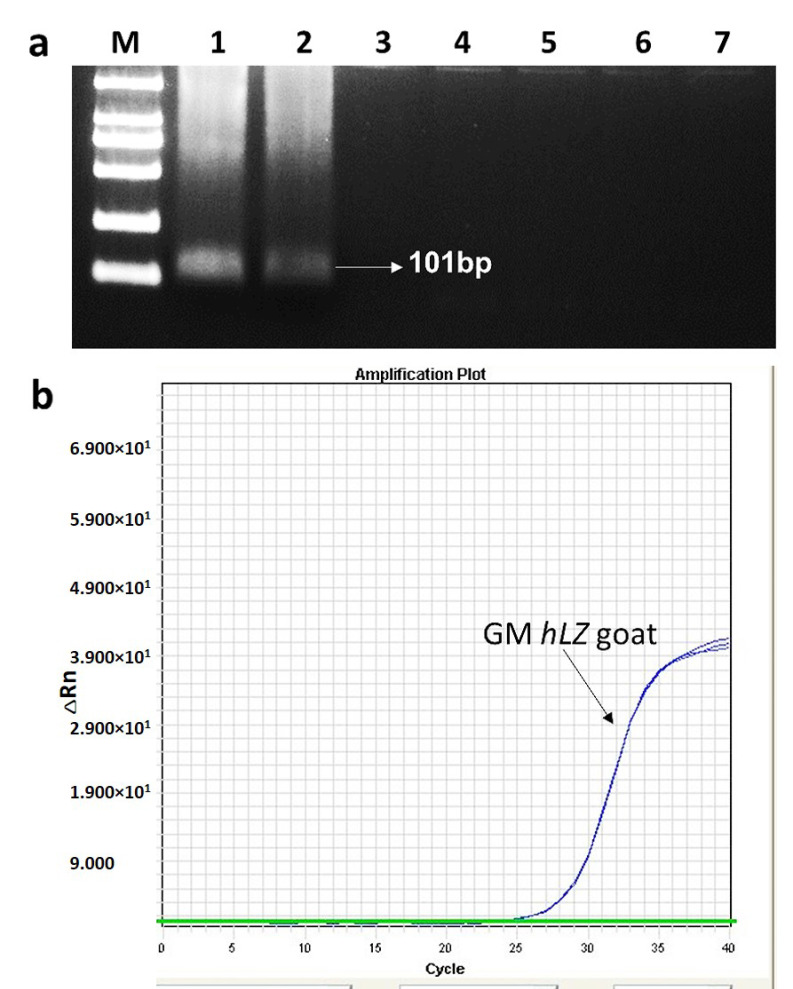
Specificity of the designed event-specific primer/probe set in conventional PCR and real-time PCR. (**a**) Agarose gel electrophoresis of conventional PCR. Lane M, DL2000 markers; lane 1–7, GM hLZ, GM hLZ, GM *hLA*, GM *hLF*, GM *hLF*, GM *hSA*, GM *hSA*, and non-GM goat lines. (**b**) Amplification curves of genomic DNA samples from GM hLZ, GM *hLF*, GM *hSA*, and non-GM goat lines.

The real-time primers and probe were designed based on the 5′ event-specific sequence of transgenic hLZ goat. The forward primer GM-LYZ-F and the GM-LYZ-P probe are located at the exogenous DNA region, and the reverse primer GM-LYZ-R is located in the goat genome. The specificity of the GM-LYZ-F/R primers were firstly tested by conventional PCR, and the results showed that only the expected DNA fragment of 101 bp in length was observed in reactions with transgenic *rhSA* lines, and no DNA amplicons or non-specific products were observed in reactions with transgenic *rhSA* lines, transgenic *rhLF* goat lines, or non-GM goats (Figure 2a).

After the real-time PCR assay was optimised, its specificity was further determined by screening different transgenic goat lines and non-GM goat lines. The results showed that a positive fluorescent signal and traditional amplification curves were only observed for reactions with transgenic hLZ goat DNA, and no fluorescent signal or traditional amplification curves were observed for reactions with transgenic *rhSA* lines, transgenic *rhLF* goat lines, or non-GM goats (Figure 2b). The results of both conventional PCR and real-time PCR indicate that the developed event-specific real-time PCR assay is highly specific for transgenic hLZ goat lines. This result was satisfied with the requests of the event-specific method according to the E.U. guidelines [22].

### 3.2. Standard Curve Construction and Dynamic Range Determination

**Figure 3:**
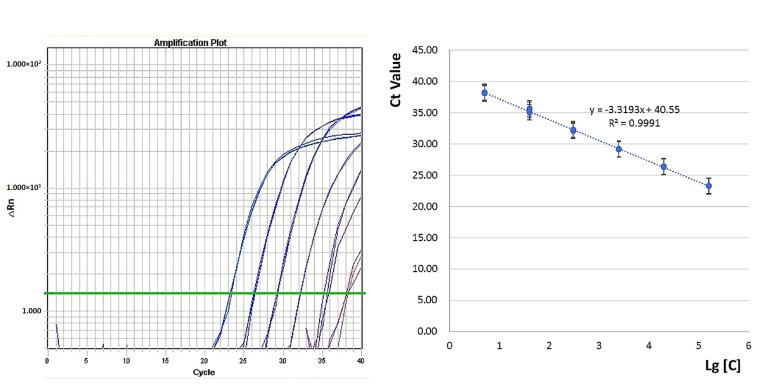
Standard curve construction and dynamic range determination of event-specific real-time PCR assay.

An accurate real-time PCR method should generate a reliable quantitative standard curve with a wide dynamic range and good linearity. For the developed event-specific assay, a standard curve was constructed using dilutions of 1.6 × 10^5^, 2 × 10^4^, 2.5 × 10^3^, 3 × 10^2^, 40 and 5 haploid genomic copies per 5 μL as calibrators. The standard curve was plotted as cycle threshold (Ct) values against the logarithm of genomic DNA copy numbers. The results revealed high PCR efficiency with a value of 1.0011 and a high square regression coefficient (*R*^2^) of 0.9991 (Figure 3). There was good linearity between DNA quantity and fluorescence (Ct) value, indicating that the event-specific assay is suitable for quantitative measurements. Based on the standard curve, the amount of each calibrator was calculated, and the quantified values were very close to the theoretical values, with a slightly low bias within 11.58%, indicating that the established event-specific real-time PCR assay has a wider dynamic range (1.6 × 10^5^ copies to 5 copies) than that of ddPCR assay [21]. The PCR efficiency and square regression coefficient of the developed assay were higher than the basic requirements of one ideal real-time PCR assay and previously published real-time PCR methods for transgenic animal detection [15,16,22].

### 3.3. LOD and LOQ Determination

In real-time PCR, LOD and LOQ are the lowest quantity of the template DNA that can be reliably detected and quantified with high accuracy at a ≥95% confidential level [22]. The absolute limit is the lowest number of initial template copies that can be detected and quantified. In order to evaluate LOD and LOQ, six DNA dilutions (2 × 10^3^, 2 × 10^2^, 10^2^, 50, 10 and 5 haploid genomic copies per reaction) were tested. As expected, the ability to detect the transgenic hLZ event-specific fragment decreased with decreasing genomic DNA copy numbers, the product was detected in all dilutions, and positive fluorescent signals and traditional amplification curves were only observed for the expected samples (Table 2). These results indicate that the LOD value was five copies according to the criterion of the European Network of GMO Laboratories (ENGL) [22]. All dilutions were accurately quantified with bias (ranging from −8.64% to 22.25%) less than 25%, except for reactions with five haploid genomic copies for which the bias was 40.78% (Table 2). Therefore, to be quantified reliably, 10 initial copies are required at least, which indicates that the LOQ of the established real-time PCR assay is 10 copies of haploid genome. The LOD and LOQ of the developed method is more sensitive than previously developed multiplex PCR assays [14]. This high level of sensitivity makes the assay suitable for samples with trace amounts of transgenic hLZ goat material.

### 3.4. Repeatability of the qPCR Assay

To validate the repeatability, standard deviation (SD) and relative standard deviation (RSD) were calculated according to the nine Ct values from three parallel experiments and three replicates. SD values ranged from 0.11 to 0.55, and RSD values ranged from 0.30% to 1.83% (Table 2). The SD and RSD values were acceptable compared with previously established real-time PCR assays [15,16], which were also below the requirements specified in the EU guideline [22]. The results indicate that the established event-specific real-time PCR assay has very good repeatability.

### 3.5. Quantitative Analysis of Simulated Blood Samples

A total of four simulated goat blood samples differing in GM content (S1–S4) were quantified using the event-specific real-time assay, and the quantified mean GM contents of S1–S4 were 5.22%, 1.91%, 0.95%, and 0.39%, respectively (Table 3). To evaluate the accuracy of the quantitative method, bias between the quantified values and the given values was calculated, and the results ranged from −4.50% to 22.00%, which were well within the acceptable range of 25% [22]. The precision was estimated by SD and RSD values, and the SD values of Ct values ranged from 0.10 to 0.42, while RSD values ranged from 0.39% to 1.30% (Table 3). These results showed that the developed event-specific real-time PCR assay satisfied the minimum E.U. performance requirements for a GMO detection method [22], with high accuracy and precision for GM content quantification, indicating that the developed method is suitable for transgenic hLZ goat detection and quantification.

### 3.6. Determination of Transgenic hLZ Goat Content in Practical Samples

Due to the impressive performance of the developed real-time PCR assay, the method was used to assess the GM content in practical samples for the risk assessment of transgenic hLZ goats. Four types of samples were prepared and tested, including 16 goat blood samples from the transgenic hLZ goat offspring (F1–F8 generations), five goat milk samples, 18 goat faecal samples, and five living environmental soil samples from areas in which transgenic goat faeces were deposited. All samples were tested using three parallel reactions, and the average Ct, SD and RSD values are shown in Table 4.

In the detection of the 16 blood samples, positive results and similar Ct values (ranging from 24.21 to 26.79) were obtained for each sample using the event-specific real-time assay, and the RSD values of all Ct values for the 16 offspring blood samples were as low as 1.96%. Positive results were also obtained in the goat species-specific assay, with all blood samples having Ct values ranging from 22.34 to 24.97. All blood samples from the eight generations were positive for the hLZ event, suggesting that the transgenic hLZ event was stably inherited (i.e., the transgenic hLZ event resulted in stable heritability of the inserted DNA). Thus, the assay could be used for future molecular characterisation of transgenic hLZ goat. Compared with the often-used methods, Southern blot hybridization and Fluorescence in situ hybridization (FISH), the developed real-time PCR assay showed the advantages of easy operation, low cost, and time saving [23].

In the detection of goat milk samples, the transgenic hLZ event content was detected in all four milk samples from transgenic hLZ goat lines (M418, M494, M502 and M540). The Ct values of these four milk samples ranged from 26.32 to 27.67, with RSD values less than 1.52% in the event-specific assay. The Ct values ranged from 24.26 to 25.19 with RSD values less than 1.15% in goat species assays. In the M-N1 milk sample from non-transgenic goats, no fluorescent signal was observed in the event-specific assay. Thus, we believe that goat milk samples contained a large amount of genomic DNA from somatic cells, and the GM content was derived from the transgenic somatic cells of transgenic hLZ goat lines. In addition, these results showed that the developed method could be used for the identification and quantification of transgenic hLZ goat content in milk and its derivatives. In GM food safety assessments, the real-time PCR method is often used to evaluate whether transgenic plant DNA fragments reside in animal tissue through feeding transgenic plant seeds or leaves. The partial DNA fragments from GM maize or rice were detected in the blood and intestinal contents [24,25]. Guo et al. (2018) observed the existence of bovine and equine DNA in milk, yogurt, and other dairy products using one triplex real-time PCR [26]. Our results also confirmed that the transgenic hLZ DNA and endogenous reference gene could be detected in fresh goat milk samples, and the developed method could be further used for the detection of the transgene content in the goat milk products.

In the analysis of the 18 fresh faecal samples, both the transgenic event and goat endogenous genes were tested using five samples (F254, F494, F502, F540, and F18014). Only goat endogenous genes were detected in two samples (F106 and F-N2), and no positive signals were observed in event-specific and goat species assays for 11 samples (F176, F350, F418, F476, F496, F498, F518, F522, F18006, F18046 and F-N1). Among the F254, F494, F502, F540 and F18014 samples, Ct values ranged from 31.15 to 33.31 in the event-specific assay, and from 27.81 to 29.57 in the goat species assay, suggesting that the amounts of event-specific and goat genome DNA were quite low. In addition, event-specific and endogenous genes were not always detected consistently among these samples. We therefore speculate that the positive results might be caused by the shedding of goat digestive tract cells, and the positive signal may not be derived from horizontal exogenous gene transfer. In the clinical prediction of cancers, DNA and protein detection methods were established by detecting the target DNA or protein from the exfoliated colonic epithelial cells, which indicated that a small number of colonic epithelial cells often shed into faecal samples, although the collection of adequate colonic epithelial cells is very difficult [27,28]. For confirming that the positive results of event and endogenous gene assays come from body cell contamination or horizontal exogenous gene transfer, next-generation sequencing tolls might be used to find the integration of partial fragment of exogenous genes. In several previous studies, similar results were also obtained. Wang et al. (2020) fed the transgenic silkworm to chicken, and no transgenic DNA fragments were detected from the chicken digesta or tissues using PCR analysis [29]. Xu et al. (2011) reported that no exogenous genes of hLZ or *hLF* were detected from the corresponding transgenic cow gut samples using both PCR and real-time PCR and concluded that no horizontal gene transfer happened between the transgenic cow and its gut microorganisms [30].

In the soil sample analysis, no positive signals or amplification curves were observed for any of the five tested samples in event-specific and goat species assays; hence, there was no event-specific goat genome DNA in soil samples from the environment in which transgenic goat faeces were deposited. Although positive results were observed for a few samples from fresh faeces of GM hLZ goat lines, genomic DNA in fresh faeces is likely degraded rapidly following deposition in environmental soil. These results might be of relevance to the risk assessment of transgenic animal excreta for assessing environmental safety. The same results were also obtained in two previous studies. Murray, D et al. (2007) evaluated the degradation time of partial DNA residence of transgenic pig carcasses in the soil environment; they found a 10^7^-fold reduction in genetic material which implied that neither transgene nor mitochondrion markers could be detectable in the soil sample [31]. Bao et al. (2015) found no transgene DNA transferred to the faeces or surrounding soils by PCR-denaturing gradient gel electrophoresis and 16S rDNA sequencing [32].

## 4. Conclusions

In conclusion, an event-specific real-time PCR assay was established based on the junction sequences surrounding the exogenous DNA integration site. The key parameters and performance of the developed assay were evaluated based on E.U. guidelines for real-time PCR methods for GMO detection. The specificity of the designed primers was validated using conventional PCR employing different transgenic goat lines and non-GM goat lines as controls. The specificity of the real-time PCR assay was further validated by employing different transgenic goat lines and non-GM goat lines as controls. The results of conventional and real-time PCR confirmed that the developed event-specific real-time PCR assay was highly specific for transgenic hLZ goat lines. The sensitivity was high, with an LOD of five copies per reaction, and an LOQ of ten copies per reaction. High accuracy and precision were confirmed using four simulated blood samples. Furthermore, the developed assay was successfully employed to test practical samples for risk assessment, including blood, milk, faecal, and soil samples. The results of blood samples from different generations showed that exogenous DNA could be inherited stably by offspring lines. The results from milk, faecal, and soil samples showed that the developed assay could quantify the GM hLZ content in trace samples, making it suitable for risk assessments, such as tracing the GM content in milk and its derivatives, release into the surrounding environment, and horizontal gene transfer.

## Figures and Tables

**Table 1:** Primers and probe for real-time polymerase chain reaction (PCR) assays.

Assays	Primer Name	Sequence (5′—3′)	Amplicon (bp)
Event-specific	GM-LYZ-F	TCTTGTCATACAGTGTACTGATAAAGC	101
	GM-LYZ-R	CTCTTCAGACCTAATAACTTCGTATAGC	
	GM-LYZ-P	FAM TGCTCCTGATAAATTAGTTCCCTTCCCACCTTTCG-BHQ1	
Goat	Goat-F	CCAACATGCCTTTAAACCCTCAA	88
	Goat-R	GGAACTGTAGCCTTCTGACTCG	
	Goat-P	FAM-TGCCTTTCCTTCCCCGCCAGTCTC-BHQ1	

**Table 2:** Limit of detection (LOD) and limit of quantification (LOQ) determination for the event-specific real-time PCR assay using different dilutions of transgenic hLZ goat DNA for calibration.

Copy Number	Ct Values				SD	RSD (%)	Quantified Copy Number	Bias (%)
	Rep1	Rep2	Rep3	Mean				
2000	30.19	29.24	29.26	29.56	0.54	1.83	2041.08	2.05
200	32.73	33.11	33.20	33.02	0.25	0.76	186.20	−6.90
100	33.95	33.83	34.35	34.04	0.27	0.80	91.36	−8.64
50	34.72	34.92	34.76	34.80	0.11	0.30	53.87	7.74
10	36.51	37.35	36.96	36.94	0.42	1.13	12.22	22.25
5	38.37	37.33	37.52	37.74	0.55	1.47	7.04	40.78

SD: standard deviation; RSD: relative standard deviation.

**Table 3:** Quantification of simulated blood samples using the developed event-specific real-time PCR assay.

Sample Name	PCR Assay	Ct Values			Mean	SD	RSD (%)	Quantified Copy Number	GM Content (%)	Bias (%)
S1 (5.0%)	Event-specific	30.87	31.31	30.79	30.99	0.28	0.90	758.80	5.22	4.40
	Goat species	24.62	25.00	24.57	24.73	0.24	0.95	14,526.36		
S2 (2.0%)	Event-specific	32.45	32.62	31.82	32.3	0.42	1.30	306.53	1.91	−4.50
	Goat species	24.42	24.56	24.77	24.59	0.18	0.72	16,036.77		
S3 (0.9%)	Event-specific	33.59	32.86	33.45	33.3	0.39	1.16	152.83	0.95	5.56
	Goat species	24.42	24.56	24.77	24.59	0.18	0.72	16,036.77		
S4 (0.5%)	Event-specific	34.87	34.31	34.79	34.66	0.30	0.87	59.63	0.39	−22.00
	Goat species	24.69	24.57	24.76	24.67	0.10	0.39	15,116.24		

**Table 4:** Detection of practical blood, milk, faecal and soil samples using the developed event-specific real-time PCR assay.

Sample Type	Sample Name	Animal Type	hLZ Event-Specific Assay			Goat Species Assay		
			Mean Ct	SD	RSD (%)	Mean Ct	SD	RSD (%)
Blood	B106	GM	24.21	0.28	1.16	22.68	0.17	0.75
	B176	GM	25.62	0.19	0.73	22.39	0.28	1.25
	B254	GM	24.45	0.18	0.72	22.81	0.27	1.18
	B350	GM	26.47	0.21	0.79	24.66	0.23	0.93
	B418	GM	25.37	0.22	0.88	23.26	0.29	1.25
	B476	GM	25.34	0.31	1.22	23.58	0.19	0.81
	B494	GM	26.59	0.22	0.84	24.32	0.18	0.74
	B496	GM	24.76	0.15	0.62	22.47	0.16	0.71
	B498	GM	24.34	0.34	1.41	22.59	0.24	1.06
	B502	GM	25.68	0.15	0.58	23.34	0.12	0.51
	B518	GM	25.47	0.29	1.14	23.37	0.19	0.81
	B522	GM	26.79	0.31	1.17	24.92	0.16	0.64
	B540	GM	25.42	0.36	1.42	23.05	0.11	0.48
	B18006	GM	26.36	0.26	0.99	24.97	0.12	0.48
	B18014	GM	25.88	0.35	1.35	23.24	0.16	0.69
	B18046	GM	25.02	0.49	1.96	23.16	0.09	0.39
Milk	M418	GM	27.67	0.35	1.26	25.14	0.29	1.15
	M494	GM	26.32	0.29	1.10	24.26	0.24	0.99
	M502	GM	27.04	0.41	1.52	25.19	0.19	0.75
	M522	GM	26.94	0.31	1.15	25.03	0.27	1.08
	M-N1	Non-GM	neg	/	/	24.96	0.18	0.72
Faeces	F106	GM	neg	/	/	32.55	1.175	3.61
	F176	GM	neg	/	/	neg	/	/
	F254	GM	33.31	0.60	1.79	29.40	0.19	0.63
	F350	GM	neg	/	/	neg	/	/
	F418	GM	neg	/	/	neg	/	/
	F494	GM	32.27	0.67	2.09	28.56	0.65	2.27
	F476	GM	neg	/	/	neg	/	/
	F496	GM	neg	/	/	neg	/	/
	F498	GM	neg	/	/	neg	/	/
	F502	GM	31.47	0.20	0.62	28.02	0.75	2.69
	F518	GM	neg	/	/	neg	/	/
	F522	GM	neg	/	/	neg	/	/
	F540	GM	33.15	0.52	1.57	29.57	0.56	1.91
	F18006	GM	neg	/	/	neg	/	/
	F18014	GM	31.48	0.38	1.21	27.81	0.20	0.71
	F18046	GM	neg	/	/	neg	/	/
	F-N1	Non-GM	neg	/	/	neg	/	/
	F-N2	Non-GM	neg	/	/	26.49	0.49	1.84
Soil	S1	GM	neg	/	/	neg	/	/
	S2	GM	neg	/	/	neg	/	/
	S3	GM	neg	/	/	neg	/	/
	S4	GM	neg	/	/	neg	/	/
	S5	GM	neg	/	/	neg	/	/

Note: “/” means no data. CT value: threshold cycle. SD: standard deviation; RSD: relative standard deviation.

## Data Availability

The datasets generated for this study are available on request to the corresponding author.

## Supplementary Materials

The following are available online at https://www.mdpi.com/article/10.3390/foods10050925/s1, Table S1: Information for practical samples.

## References

[1] Miao X. (2013). Recent advances in the development of new transgenic animal technology. Cell Mol. Life Sci..

[2] Van Veen H.A., Koiter J., Vogelezang C.J., Van Wessel N., Van Dam T., Velterop I., Van Houdt K., Kupers L., Horbach D., Salaheddine M. (2012). Characterization of recombinant human C1 inhibitor secreted in milk of transgenic rabbits. J. Biotechnol..

[3] Ledford H. (2015). Salmon approval heralds rethink of transgenic animals. Nature.

[4] CAC (2008). Guideline for the Conduct of Food Safety Assessment of Foods Derived from Recombinant-DNA Animals.

[5] Moghissi A.A., Jaeger L.M., Shafei D., Bloom L.L. (2018). Regulatory science requirements of labeling of genetically modified food. Crit. Rev. Biotechnol..

[6] Alarcon C.M., Shan G.M., Layton D.T., Bell T.A., Whipkey S., Shillito R.D. (2019). Application of DNA and Protein Based Detection Methods in Agricultural Biotechnology. J. Agric. Food Chem..

[7] Salisu I.B., Shahid A.A., Yaqoob A., Ali Q., Bajwa K.S., Rao A.Q., Husnain T. (2017). Molecular Approaches for High Throughput Detection and Quantification of Genetically Modified Crops: A Review. Front. Plant. Sci..

[8] Kamle S., Ali S. (2013). Genetically modified crops: Detection strategies and biosafety issues. Gene.

[9] Zhang D., Guo J. (2011). The development and standardization of testing methods for genetically modified organisms and their derived products. J. Integr. Plant. Biol..

[10] Huang J., Wang A., Huang C., Sun Y., Song B., Zhou R., Li L. (2020). Generation of marker-free pbd-2 knock-in pigs using the CRISPR/Cas9 and Cre/loxP systems. J. Genes..

[11] Chen X.Y., Zhu Z.W., Yu F.X., Huang J., Hu X.R., Pan J.Z. (2016). Production of germline transgenic pigs co-expressing double fluorescent proteins by lentiviral vector. J. Anim. Reprod. Sci..

[12] Li X.L., Wang Q., Zhu Z., Wu S.Q., Qiu S.Y., Liu X.F., Sun M., Pan D.K., Lin X.M. (2015). Establishment of multiplex PCR for detection of myostatin knockout pigs. J. Nanjing Agric. Univ..

[13] Yang X., Cheng Y.T., Tan M.F., Zhang H.W., Liu W.Q., Zou G., Zhang L.S., Zhang C.Y., Deng S.M., Yu L. (2015). Over expression of porcine beta-defensin 2 enhances resistance to *actinobacillus pleuropneumoniae* infection in pigs. J. Infect. Immun..

[14] Liu X.F., Qiu S.Y., Li X.L., Liu D.D., Jing H.L., Wang Q., Lin X.M., Pan D.K., Shi N.N. (2018). Establishment of a Decaplex PCR-Capillary Gel Electrophoresis Method for the Simultaneous Detection of Six Kinds of Genetically Modified Animals. J. AOAC Int..

[15] Hafsa A.B., Nabi N., Zellama S.M., Said K., Chaouachi M. (2016). A new specific reference gene based on growth hormone gene (GH1) used for detection and relative quantification of Aquadvantage GM salmon (*Salmo salar* L.) in food products. Food Chem..

[16] Nakajima O., Nakamura K., Kondo K., Akiyama H., Teshima R. (2013). Method of detecting genetically modified chicken containing human erythropoietin gene. Biol. Pharm. Bull..

[17] Ragland S.A., Criss A.K. (2017). From bacterial killing to immune modulation: Recent insights into the functions of lysozyme. PLoS. Pathog..

[18] Gui T., Zhang M., Chen J.W., Zhang Y.L., Zhou N., Zhang Y., Tao J., Sui L.C., Li Y.S., Liu Y. (2012). In vitro evaluation of a mammary gland specific expression vector encoding recombinant human lysozyme for development of transgenic dairy goat embryos. Biotechnol. Lett..

[19] Maga E.A., Shoemaker C.F., Rowe J.D., Bondurant R.H., Anderson G.B., Murray J.D. (2006). Production and processing of milk from transgenic goats expressing human lysozyme in the mammary gland. J. Dairy Sci..

[20] Yu H., Chen J.Q., Liu S., Zhang A., Xu X.J., Wang X.B., Lu P., Cheng G.X. (2013). Large-scale production of functional human lysozyme in transgenic cloned goats. J. Biotechnol..

[21] Wang Q., Cai Y., He Y., Yang L., Pan L. (2018). Droplet digital PCR (ddPCR) method for the detection and quantification of goat and sheep derivatives in commercial meat products. Eur. Food Res. Technol..

[22] Marchiesi U., Mazzara M., Broll H., Giacomo D.M., European Network of GMO Laboratories (2015). Definition of minimum performance requirements for analytical methods of GMO testing. http://gmo-crl.jrc.eceuropa.eu/doc/Mm_PerfRequirements_Analyticalmethods.pdf.

[23] Waigmann E., Paoletti C., Davies H., Perry J., Kärenlampi S., Kuiper H. (2012). Special issue: Risk assessment of Genetically Modified Organisms (GMOs). EFSA J..

[24] Nawaz M.A., Mesnage R., Tsatsakis A.M., Golokhvast K.S., Yang S.H., Antoniou M.N., Chung G. (2019). Addressing concerns over the fate of DNA derived from genetically modified food in the human body: A review. Food Chem. Toxicol..

[25] Sharma R., Damgaard D., Alexander T.W., Dugan M.E., Aalhus J.L., Stanford K., McAllister T.A. (2006). Detection of transgenic and endogenous plant DNA in digesta and tissues of sheep and pigs fed Roundup Ready canola meal. J. Agric Food Chem..

[26] Guo L., Qian J.-P., Guo Y.-S., Guo X.H., Liu -Q., Luo J.X., Ya M. (2018). Simultaneous identification of bovine and equine DNA in milks and dairy products inferred from triplex TaqMan real-time PCR technique. J. Dairy Sci.

[27] Yu Y.J., Majumdar A.P., Nechvatal J.M., Ram J.L., Basson M.D., Heilbrun L.K., Kato I. (2008). Exfoliated cells in stool: A source for reverse transcription-PCR-based analysis of biomarkers of gastrointestinal cancer. Cancer Epidemiol. Biomark. Prev..

[28] Anderson N., Suliman I., Bandaletova T., Obichere A., Lywood R., Loktionov A. (2011). Protein biomarkers in exfoliated cells collected from the human rectal mucosa: Implications for colorectal disease detection and monitoring. Int. J. Colorectal. Dis..

[29] Wang Y., Wang Z., Guo H., Huang J., Li X., Sun Q., Wang B., Xie E., Jiang L., Xia Q. (2020). Potential of transferring transgenic DNA from silkworm to chicken. Int. J. Biol. Macromol..

[30] Xu J., Zhao J., Wang J., Zhao Y., Zhang L., Chu M., Li N. (2011). Molecular-based environmental risk assessment of three varieties of genetically engineered cows. Transgenic Res..

[31] Murray D., Meidinger R.G., Golovan S.P., Phillips J.P., O’Halloran I.P., Fan M.Z., Hacker R.R., Forsberg C.W. (2007). Transgene and mitochondrial DNA are indicators of efficient composting of transgenic pig carcasses. Bioresour Technol..

[32] Bao Z., Gao X., Zhang Q., Lin J., Hu W., Yu H., Chen J., Yang Q., Yu Q. (2015). The Effects of GH Transgenic Goats on the Microflora of the Intestine, Feces and Surrounding Soil. PLoS ONE.

