# Supplementary material for: An Event-Specific Real-Time PCR Method for Measuring Transgenic Lysozyme Goat Content in Trace Samples
Source: Foods. 2021 Apr 23;10(5):925. doi: 10.3390/foods10050925 (PMC8146569; doi:10.3390/foods10050925)
Supplement: Supplementary file 1 [file foods-10-00925-s001.zip › foods-1119567-supplementary.pdf]

Supplementary Table S1. Information for practical samples

Sample type	Sample name	Animal type	Goat code number	Goat generation
Blood	B106	GM	106	F1
	B176	GM	176	F2
	B254	GM	254	F2
	B350	GM	350	F3
	B418	GM	418	F3
	B476	GM	476	F4
	B494	GM	494	F4
	B496	GM	496	F4
	B498	GM	498	F5
	B502	GM	502	F5
	B518	GM	518	F6
	B522	GM	522	F6
	B540	GM	540	F7
	B18006	GM	18006	F7
	B18014	GM	18014	F8
	B18046	GM	18046	F8
Milk	M418	GM	418	F3
	M494	GM	494	F4
	M502	GM	502	F5
	M522	GM	522	F6
	M-N1	Non-GM	AB-1	
Faeces	F106	GM	106	F1
	F176	GM	176	F2
	F254	GM	254	F2
	F350	GM	350	F3
	F418	GM	418	F3
	F494	GM	494	F4

	F476	GM	476	F4
	F496	GM	496	F4
	F498	GM	498	F5
	F502	GM	502	F5
	F518	GM	518	F6
	F522	GM	522	F6
	F540	GM	540	F7
	F18006	GM	18006	F7
	F18014	GM	18014	F8
	F18046	GM	18046	F8
	F-N1	Non-GM	AB-1	/
	F-N2	Non-GM	AB-2	/
	S1	GM	/	/
	S2	GM	/	/
Soil	S3	GM	/	/
	S4	GM	/	/
	S5	GM	/	/
